# Draft Genome Sequence of *Lactobacillus fermentum* NCDC 400, Isolated from a Traditional Indian Dairy Product

**DOI:** 10.1128/genomeA.01492-17

**Published:** 2018-01-11

**Authors:** Syed Azmal Ali, Sudarshan Kumar, Ashok Kumar Mohanty, Pradip Behare

**Affiliations:** a Proteomics and Cell Biology Lab, Animal Biotechnology Center, ICAR, National Dairy Research Institute, Karnal, Haryana, India; b National Collection of Dairy Cultures (NCDC) Lab, Dairy Microbiology Division, ICAR, National Dairy Research Institute, Karnal, Haryana, India

## Abstract

We announce here the draft genome sequence of *Lactobacillus fermentum* NCDC 400, a potential probiotic strain isolated from a traditional Indian dairy product. The genome size of *Lactobacillus fermentum* NCDC 400 is 1.89 Mb, and the assembled sequence consists of 185 contigs joined into 138 scaffolds.

## GENOME ANNOUNCEMENT

The species *Lactobacillus fermentum* is a Gram-positive facultative heterofermentative rod belonging to the *Firmicutes* phylum (1). It is a natural inhabitant of the human gastrointestinal tract (GIT) and often isolated from dairy and nondairy sources. While some strains are used as probiotics, others are commonly employed as a starter culture during food fermentation processes. The uniqueness of certain strains is to endure the harsh GIT conditions, such as high concentrations of acid and bile, to transiently inhabit the host. Recently, published studies emphasized that the intake of lactobacilli helps improve gut homeostasis in the host and irritable bowel syndrome (IBS) symptoms (2–7).

*Lactobacillus fermentum* strain NCDC 400, an indigenous isolate from the Indian dairy product dahi, was identified using 16S rRNA (GenBank accession number MF289554). This strain has the ability to produce a great amount of exopolysaccharides (EPS), which helps this bacterium to improve technological properties of low-fat fermented dairy products (8). The strain exhibits probiotic attributes, such as cell surface hydrophobicity, inhibition of pathogen adherence to gut epithelial cell lines, such as Caco-2 and HT29 cells, bile salt and high acid tolerance, cellular autoaggregation, antibacterial activity, antioxidative activity, and *in vivo* immune modulation. It has also been found to upregulate *S*-adenosylmethionine synthetase (SAM) in the presence of bile stress to counteract its hazardous effect (9). The molecular mechanisms of the putative probiotic are scanty; one of the mechanisms to elucidate the host-microbe interactions is the analysis of genomic data (10).

The present study utilized paired-end sequencing using the Illumina HiSeq 2500 platform and identified a total of 17,733,506 high-quality (HQ) reads by Velvet, KmerGenie, SSPACE, and GapFiller. The final assembly of 138 scaffolds, with an *N*_50_ of 27,950 bp, maximum scaffold size of 101,072 bp, and genome size of 1.89 Mb, showed the relative genome length in comparison with those of other sequenced *L. fermentum* strains (10–13). Further, GeneMarkS predicted 1,996 protein-coding genes in the total scaffolds, and these proteins were annotated using BlastP against the nonredundant database of NCBI, with an E value of <1e-6, and 1,961 of these genes were found to be homologous to *Lactobacillus fermentum* proteins. A total of 1,286 GO terms involved in biological processes, 423 GO terms in cellular components, and 1,466 GO terms in molecular functions were assigned. These genes were also annotated using KO terms (or pathway annotation) using the KAAS-KEGG Web server, wherein 594 genes were annotated.

Finally, a total of 56 tRNA genes in the genome were predicted using tRNAscan-SE. Also, a total of 15 rRNA genes (5 5S rRNAs, 4 16S rRNA, and 6 23S rRNAs) were found by BLASTN, and the scaffolds were aligned against the Rfam database. The whole draft genome result suggests that the identified genome is involved in metabolism processes, genetic and environmental information processing, and cellular processes which assist in bacterial tolerance toward various gastrointestinal challenges.

### Accession number(s).

This whole-genome shotgun project has been deposited at DDBJ/ENA/GenBank under the accession number PDKX00000000. The version described in this paper is version PDKX01000000.
